# Retraction notice to “The mouse complement regulator CD59b is significantly expressed only in testis and plays roles in sperm acrosome activation and motility” [Mol. Immunol. 45 (2008) 534–542]

**DOI:** 10.1016/j.molimm.2013.12.001

**Published:** 2014-03

**Authors:** Rossen M. Donev, Baalasubramanian Sivasankar, Masashi Mizuno, B. Paul Morgan

**Affiliations:** Department of Medical Biochemistry and Immunology, School of Medicine, Cardiff University, Cardiff CF14 4XN, UK

This article has been retracted: please see Elsevier Policy on Article Withdrawal (http://www.elsevier.com/locate/withdrawalpolicy).

This article has been retracted at the request the Editor following the release of the conclusions of an internal investigation panel established by Cardiff University to examine allegations of research misconduct in the preparation of the manuscript. The panel found evidence of splicing or pasting affecting Figures 1B, 4A and 5, without indication that this had been done. While these image manipulations cannot be characterized as “fabrication” because there is no reason to doubt the validity of the underlying science in the article, they represent unacceptable practice when submitting a manuscript for publication. The panel concluded that Dr. Donev was solely responsible for these actions and that none of the other co-authors of this manuscript knew, or had reason to suspect, that the data presented in the manuscript had been manipulated by Dr. Donev.

